# Peer victimization and children’s internet addiction in China: a moderated mediation model

**DOI:** 10.3389/fpsyg.2023.1236135

**Published:** 2023-10-20

**Authors:** Pingyan Zhou, Jinping Cai, Jiaxin Cui, Jian Liu, Wenguang He, Cai Zhang, Fumei Chen, Zhe Wang

**Affiliations:** ^1^School of Psychology, Qufu Normal University, Qufu, Shandong Province, China; ^2^Collaborative Innovation Center of Assessment for Basic Education Quality, Beijing Normal University, Beijing, China; ^3^School of Psychology, Shenzhen University, Shenzhen, Guangdong Province, China; ^4^College of Education, HeBei Normal University, Shijiazhuang, HeBei Province, China

**Keywords:** peer victimization, internet addiction, subjective well-being, parent–child relationship, moderated mediation model

## Abstract

**Background:**

Peer victimization used to be considered as a crucial risk factor for children addicted to the internet. Whereas some victimized ones are function better than would be expected. Variability across individuals indicates that it is necessary to understand how children cope with being bullied and why they do not exhibit maladaptive outcomes.

**Objective:**

We explored the underlying mechanisms by testing whether subjective well-being was a mediator between peer victimization and Internet addiction and whether the mediation effects conditioned on the levels of parent–child relationship (PCR).

**Methods:**

Data were collected from 65, 868 elementary school students in China (Mage = 9.56 years, SD = 0.62, 54.0% male) using four questionnaires.

**Results:**

We found that: (1) subjective well-being can partially mediate the relationship of the two variables; and (2) PCR can moderate direct path and second half of the intermediary process. These moderating effects were stronger for children with higher PCR vs. lower PCR, as a strong PCR can help children to deal with intense emotions and to access effective resources to obtain support.

**Conclusion:**

This study deepens our understanding of how peer victimization leads to internet addiction, identifies a supportive PCR as a crucial factor that strengthens the resilience of child victims, and highlights the value of focusing on improving the relationship between parents and children in intervening internet addiction related to peer victimization.

## 1. Introduction

Internet addiction (IA) refers to a disorder associated with difficulties in controlling the impulse of using the internet, namely, compulsive internet use (Young, 1996). Studies showed that Internet addiction was multidimensional with different neurobiological underpinnings (Dong et al., 2012; Tahir et al., 2021). For example, Tahir et al. demonstrated that Internet addiction included 5 dimensions: excessive use, neglect work, anticipation, lack of control, neglect social life (Tahir et al., 2021). Internet addiction is evolving. Individuals infected with Covid-19 have higher levels of IA (Tahir et al., 2021). In addition, it includes multiple subtypes, such as cyber relationship addiction, and Internet compulsion (De Berardis et al., 2009). The incidence of problematic internet use is rising and the age of addiction has decreased (Li et al., 2014; Zhou et al., 2017). Internet addiction has widespread ramifications of sleep disorders, academic problems, poor interpersonal skills (Young, 2004; Anderson et al., 2017), and mental disorders (e.g., depression) (Han et al., 2017), and even has grave, long lasting adverse effects on psychosocial adjustment (Muusses et al., 2014; Ciarrochi et al., 2016), suggesting that intervening to IA is of great significance. Until now, many methods have been adopted to alleviate individuals’ addiction behaviors, however, research on prevention and intervention programs for addressing this phenomenon is still in its early stages (Vondrackova and Gabrhelik, 2016; Lopez-Fernandez and Kuss, 2020).

Peer related factors play a vital affective role in children’s development (DeSteno et al., 2013). One of these factors, peer victimization, being attacked by one or more students with intention repeatedly and over time, has drawn extensive scholarly attention (Olweus, 1993; Sheppes et al., 2011; McRae and Gross, 2020). Peer victimization can be a prominent stress source for individuals and has a high prevalence rate (10–35%) (Moore et al., 2017). Further, peer victimization is strongly tied to IA (Strittmatter et al., 2014; Hsieh et al., 2016, 2019). With a sample of 2,758 Chinese middle school students (*M*age = 13.56), Zhai et al. revealed that being bullied by peers was positively related to IA (Zhai et al., 2019). This can be explained by the self-medication hypothesis (Hsieh et al., 2016), which posits that abuse in the internet may be a strategy individuals adopted to avoid or escape negative emotions induced by traumatic experiences or life stress. Namely, IA is a non-adaptive strategy that can decrease mental pressure and emotional disorders resulting from the experience of victimization. When children suffer from peer victimization, their relationships with their peers are at their most dysfunctional, putting them at risk for developing emotional disturbances or mood disorders (Reijntjes et al., 2010). Then, children being bullied by their peers might lose themselves in cyberspace to avoid intensive maladaptive emotions to feel a sense of comfort, ultimately, putting themselves at high risk of Internet addiction (Reijntjes et al., 2011; Boniel-Nissim and Sasson, 2018; Hsieh et al., 2019). Additionally, peer victimization not only physically harms children, but also reduces their sense of belonging, thereby threatening their basic needs. The compensatory satisfaction theory posits that when psychological needs are not met in reality, children will seek satisfaction through the Internet, which can lead to Internet addiction (Suler, 1999; Liu et al., 2016). Although the direct relationships linking peer victimization and IA have been verified (Jia et al., 2018; Hsieh et al., 2019), few people know about the mediation mechanism (how) and the moderation mechanism (under what conditions) that peer victimization is related to Internet addiction. These factors are necessary to be explored to develop targeted intervention methods to reduce children’s Internet addiction.

### 1.1. Subjective well-being as a mediator

With the rise of positive psychology, subjective well-being, regarded as its key component, has also drawn attention. Subjective well-being refers to one’s self-evaluation about their life quality and inner feelings across various domains, including cognitive states and ongoing affect (Diener et al., 1999). Cognitive well-being is related to self-judgement about one’s life satisfaction both globally and across various domains (such as friends and school), while emotional well-being refers to more frequent experiences of positive (vs. negative) affect (Diener et al., 1999). Being bullied by peers is a strong risk factor of lower subjective well-being for kids, teenagers, and young persons (Guhn et al., 2013; Tiliouine, 2015; Mahuteau and Zhu, 2016; Kaakinen et al., 2018). A study with samples from 15 countries, even controlled numerous confounding variables (e.g., age, geographic region), children’s subjective well-being was negatively related to physical and psychological bullying (Savahl et al., 2019). Likewise, children who were chronically bullied reported the worst emotional well-being compared to those who were not (Yubero et al., 2021). As a meta-analysis showed, children being bullied earlier have greater probability to exhibit lower subjective well-being seven years later, suggesting that experiences of peer victimization have long lasting effect on one’s subjective well-being (Gini et al., 2014). Goswami established that the best two predictors of subjective well-being in children were having good relationships with their peers and family members (Gross, 1999). On the one hand, if relationships with their peers are damaged by peer victimization, children’s subjective well-being will be harmed. On the other hand, due to their psychological need for autonomy and independence, children are less likely to seek help from significant adults in their lives. Thus, children being bullied may be at greater risk of decreased subjective well-being than their peers not being bullied due to lacking family supports. In prior research, when individuals were bullied for a period of time, their need for a sense of belonging could not be fulfilled, resulting in more negative emotions and leading to decreased subjective well-being (Gross, 2001, 2015).

Subjective well-being strongly predicted Internet addiction (Agbaria and Bdier, 2020). Compared to people with higher levels of SWB, those with lower levels may react to emotional stimulus more slowly and tackle them with non-adaptive strategies, in turn resulting in more negative feelings and less satisfying relationships (Diener and Diener, 1996; Agbaria and Bdier, 2020). According to the Psychological Decompensation Hypothesis (Mauss et al., 2007), when children’s psychological needs from peer relationships are not well met in real life, they will turn to other ways to seek satisfaction, such as the internet. Once their needs are met by the internet, going online will be reinforced and strengthened continuously, and eventually they will become addicted to the internet. Studies found that subjective well-being were negatively associated with the risky of Internet addiction (Nie et al., 2017; Suresh et al., 2018; Agbaria and Bdier, 2020). For instance, those who experiencing decreased subjective happiness were deemed themselves more vulnerable to Internet addiction (Suresh et al., 2018). Thus, we suppose that subjective well-being is a mediator between peer victimization and IA.

### 1.2. Parent–child relationship as a moderator

Although internet addiction could be generated through lasting peer victimization, the different effects among individuals indicate that several protective factors keep them away from adverse consequences. Parent–child relationship (PCR) encompasses interactions that occur between parents and their offspring. It sets the foundation to establish other future interpersonal ties for children (McDowell and Parke, 2009). When bullying occurs, children may be protected from its deleterious effects on their mental health, since their relationships with their caregivers are characterized by effective communication, mutual acceptance, trust, and closeness (Rudolph et al., 2020). According to the protective model of resilience (Fergus and Zimmerman, 2005), on the one hand, the negative impact of risk factors on outcomes can be mitigated by protective factors; on the other hand, children are more immune to adversities because of these factors (Hinduja and Patchin, 2017). According to the model, PCR, as a protective factor, may dampen the side effects of peer victimization on Internet addiction, and strengthen the positive effects of subjective well-being on Internet addiction. Since children with supportive PCR can learn interpersonal skills and management experiences from their family and apply these rules to other interactive situations (Parke and Ladd, 1992; Healy et al., 2015), this increases their ability to adjust emotionally and behaviorally to resolve interpersonal conflicts (Bowes et al., 2010). Further, warm family bonds and supportive family environments could better enable children to regulate negative cognition and emotions (Rudolph et al., 2020), thereby reducing incidence of IA. Conversely, children with poor PCR incline to go online to avoid interpersonal conflicts and negative moods without enough internalizing and externalizing resources (Etkin et al., 2015), ultimately leading to internet addiction. The impacts of parenting style on one’s internalizing and externalizing problems was conditional on the quality of PCR (Steele and McKinney, 2019). Thus, it can be speculated that PCR could moderate the direct and indirect pathways of the mediation process.

### 1.3. The present study

Guided by the protective model of resilience, we aimed to investigate how Internet addiction was affected by peer victimization and under what conditions the mediating process was most potent among children. This work extends previous literature to explore the established relationships to elucidate the potential mechanisms. We have proposed three hypotheses based on the above summary: (1) Subjective well-being would be a mediator between peer victimization and IA; (2) Interactions of peer victimization and PCR would predict Internet addiction, and the interactions would be more powerful when PCR was higher vs. lower, and (3) Interactions of subjective well-being and PCR would predict Internet addiction, and the interactions would depend on the levels of PCR. A moderated mediation model would be constructed referring to the three questions.

## 2. Materials and methods

### 2.1. Participants

Participants were elementary school students, recruited in 330 urban elementary schools in Shijia Zhuang and Zheng Zhou Cities of China. In Zhengzhou, the students were from all the city’s 280 urban elementary schools, equivalent to the total numbers of primary-school students in the fourth grade in this region. In Shijiazhuang, a three-stage sampling design was adopted to select students. First, we randomly chose 10 districts to reduce sampling bias. Second, a total of 50 schools from the 10 districts were picked out randomly. Third, we chose all fourth-grade students from the 50 schools. In total, the sample included 65,868 fourth-grade children (54.0% male, *M*age = 9.56 years, SD = 0.62). The age range is 8 ~ 12 years old. We obtained written consents from all students and oral consents from their parents and teachers. In their classrooms, teachers explained the survey’s purpose to the students and administered four questionnaires to be completed during the period from September to October 2015. All participants volunteered to take part in the survey. Everyone was explicitly notified that their answers will be kept confidential.

All participants were right-handed, had normal corrected vision, and had no history of mental illness. A total of 65,868 students were approached and 64,703 valid questionnaires were recovered. Data beyond ±3 standard deviations (1,165) for subjective well-being and PCR were deleted from the original cases. The deleted data accounted for 1.8% of the total data. All four main variables and gender had a missing ratio of less than 0.7%. The missing rate for family socioeconomic status is 16.4%. All missing data (12, 244) were interpolated by regression method.

### 2.2. Measures

We revised four questionnaires on the basis of the translated Chinese versions. The revision process as follows: (1) We revised or removed some items based on interviews with the target population; (2) To compare differences between the two versions of questionnaires (English and Chinese), they were translated back into English before the pre-test; and (3) We computed the psychometric indexes of the revised questionnaires with the pre-test data. All psychometric indicators met the psychometric criteria. In the pre-test, for the Questionnaire of peer victimization, the Cronbach’s alpha coefficient was 0.88. The confirmatory factor analysis (CFA) results displayed acceptable fit indices, *χ*^2^/df = 1146.09, CFI = 0.93, TLI = 0.89, RMSEA = 0.13. For the Internet Addiction Scale, the Cronbach’s alpha coefficient was 0.81. The CFA results indicated acceptable fit indices, *χ*^2^/df = 215.72, CFI = 0.95, TLI = 0.93, RMSEA = 0.06. For the Subjective Well-being Scale, the Cronbach’s alpha coefficient was 0.93. The CFA results showed acceptable fit indices, *χ*^2^/df = 1676.79, CFI = 0.89, TLI = 0.85, RMSEA = 0.16. For the Parent–Child Relationship Scale, the Cronbach’s alpha coefficient was 0.78. The CFA results indicated acceptable fit indices, *χ*^2^/df = 744.10, CFI = 0.87, TLI = 0.83, RMSEA = 0.11.

#### 2.2.1. Peer victimization

We measured peer victimization with the Olweus Bully/Victim Questionnaire (Olweus, 1993), which contained 7 items to assess the frequency of physical, verbal, and relational forms of bullying experienced by children (e.g., “Have you ever been teased by other kids at school?”). Each item has 5 options (0 times, 1 times, 2 times, 3 ~ 4 times, more than 5 times). The higher the score, the greater the frequency of victimization by their peers. The Cronbach’s alpha coefficient of this study was 0.892. The CFA results indicated acceptable fit indices, χ^2^/df = 1146.086, CFI = 0.93, TLI = 0.89, SRMR = 0.04, RMSEA = 0.13.

#### 2.2.2. Internet addiction

We measured children’s Internet addiction with the Internet Addiction Diagnostic Questionnaire, which was developed by Young (1998) and translated into the Chinese version by Mao and colleagues (Young et al., 2000). The scale contained ten “yes” or “no” questions to measure an individual’s impulse control, tolerance, negative consequences, and withdrawal symptoms. Participants who answered “yes” to 5 or more of the 10 questions were classified as having a tendency towards Internet addiction. The Cronbach’s alpha coefficient of this study was 0.816. The CFA results demonstrated good fit indices, χ^2^/df = 275.964, CFI = 0.96, TLI = 0.93, SRMR = 0.03, RMSEA = 0.07.

#### 2.2.3. Subjective well-being

A total of 9 items were contained in the Subjective Well-being Scale, which was revised from the Index of Well-being Scale (Campbell et al., 1976). It mainly assesses one’s current level of happiness. Participants were required to answer each of the 9 items on a 7- point Likert scale (1 = definitely match; 7 = definitely not match). The scale includes two dimensions: the overall emotion index (8 items) and the life satisfaction (1 item). The total score was the mean score of 9 items, with a weight of 1 for emotion and a weight of 1.1 for life satisfaction. A higher score indicated a greater level of subjective well-being. The Cronbach’s alpha of the scale was 0.811. The CFA results indicated acceptable fit indices, χ^2^/df = 1676.786, CFI = 0.89, TLI = 0.85, SRMR = 0.05, RMSEA = 0.16.

#### 2.2.4. Parent–child relationship

The Parent–child Relationship Scale was revised from the Network Relationships Inventory (Furman and Buhrmester, 1992), which contained 11 items and reflected the relationships between one and his/her significant others. All participants were asked to rate each of the 11 items (e.g., “Do you and your parents feel annoyed with each other?”) with four options (1 = never; 2 = occasionally; 3 = sometimes; 4 = often). The total score was the average of 11 items. The higher the score, the better the PCR quality. The Cronbach’s alpha was 0.775 for this study. The CFA results showed acceptable fit indices, χ^2^/df = 744.096, CFI = 0.87, TLI = 0.83, SRMR = 0.06, RMSEA = 0.10.

#### 2.2.5. Socioeconomic status (SES)

SES was reported by students on parents’ occupation, parents’ educational level, and family possessions. Parents’ occupation was calibrated according to occupational classification, which includes 8 grades. Parents’ educational level includes 7 grades. There are five categories of family possessions. The highest grades of parents’ occupation and educational level were standardized. The sum of all family possessions were also standardized. The score of SES is the average of the three standardized scores. The Cronbach’s alpha for the inquiry was 0.571 in this study.

### 2.3. Data collection and analysis

The statistical analysis were performed with SPSS 26.0 and Mplus 7.0. We set the significance level at *p* < 0.05. All continuous variables in the current study were standardized. We analyzed the data in three steps. Firstly, CFA were adopted to verify validities of the four scales, reflected by the factor structures and indexes. The CFA results indicated good fit of the data for all four scales, and all four scales had good reliability. In the second step, the correlations between five variables of peer victimization, Internet addiction, subjective well-being, PCR, and gender were computed. Finally, we constructed a moderated mediation model with PROCESS macro (Model 15) (Hayes, 2013) to examine whether the mediating effects of subjective well-being on the association between peer victimization and Internet addiction were depended on the different levels of PCR, with gender and SES as covariate variables.

## 3. Results

### 3.1. Descriptive information and bivariate analysis

The means and standard deviations of six main variables were calculated (Table 1). The correlation analysis revealed that Internet addiction was negatively correlated with PCR, subjective well-being, SES, and gender, and positively correlated with peer victimization. Subjective well-being was positively linked to PCR, SES, and gender, and negatively linked to peer victimization. And peer victimization was negatively related to PCR, SES, and gender. PCR was positively linked to SES and gender. SES was also positively linked to gender. All five variables showed significant gender differences with independent-sample t-tests.

**Table 1:** Descriptive statistics and correlations between six variables.

Variables	1	2	3	4	5	6
Gender	1					
Internet addiction	−0.257^**^	1				
Subjective well-being	0.041^**^	−0.210^**^	1			
Peer victimization	−0.155^**^	0.310^**^	−0.285**	1		
Parent-child relationship	0.102^**^	−0.365^**^	0.408**	−0.380**	1	
Socioeconomic status (SES)	0.033^**^	−0.139^**^	0.191^**^	−0.113^**^	0.198^**^	1
Means	1.460	2.244	11.844	0.929	3.344	0.014
Standard deviation (SD)	0.498	2.459	2.853	0.994	0.499	0.732

^**^p< 0.01.

### 3.2. Testing for mediation effect

**Figure 1:**
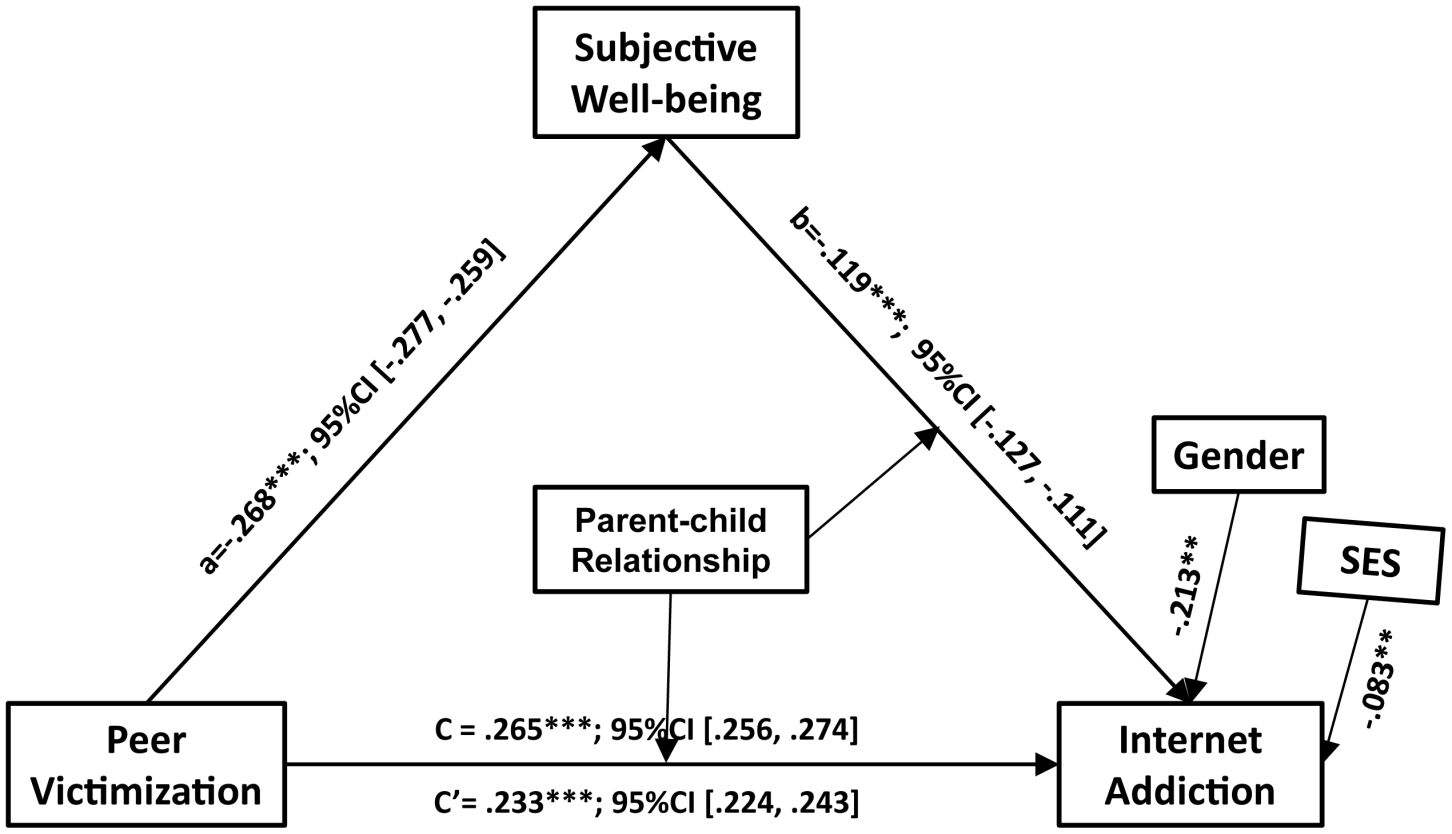
The association between peer victimization and IA was partially mediated by subjective well-being. The letter “c” represents the overall effect of peer victimization on IA. The letter “c’” represents the direct effect of peer victimization on IA. Gender was a controlling variable. IA, Internet addiction. ^***^p < 0.001, ^**^p < 0.01.

The overall effect of peer victimization on Internet addiction was significant (*β* = 0.265, 95% CI = [0.256, 0.274]). After controlling for indirect effects, the regression coefficient (direct effect) of peer victimization on Internet addiction was still significant (*β* = 0.233, 95% CI = [0.224, 0.243]). Peer victimization was negatively related to subjective well-being (*β* = −0.268, 95% CI = [−0.277, −0.259]) and subjective well-being was significantly associated with Internet addiction (*β* = −0.119, 95% CI = [−0.127, −0.111]) (Figure 1). The results suggested that subjective well-being partially mediated the relationship between peer victimization and Internet addiction. The ratio of indirect to the total effects (effect size) was 12.03%, indicating that within effects of peer victimization on Internet addiction, 12.03% passed through the mediating pathway of subjective well-being.

### 3.3. The moderating effect of parent–child relationship

**Figure 2:**
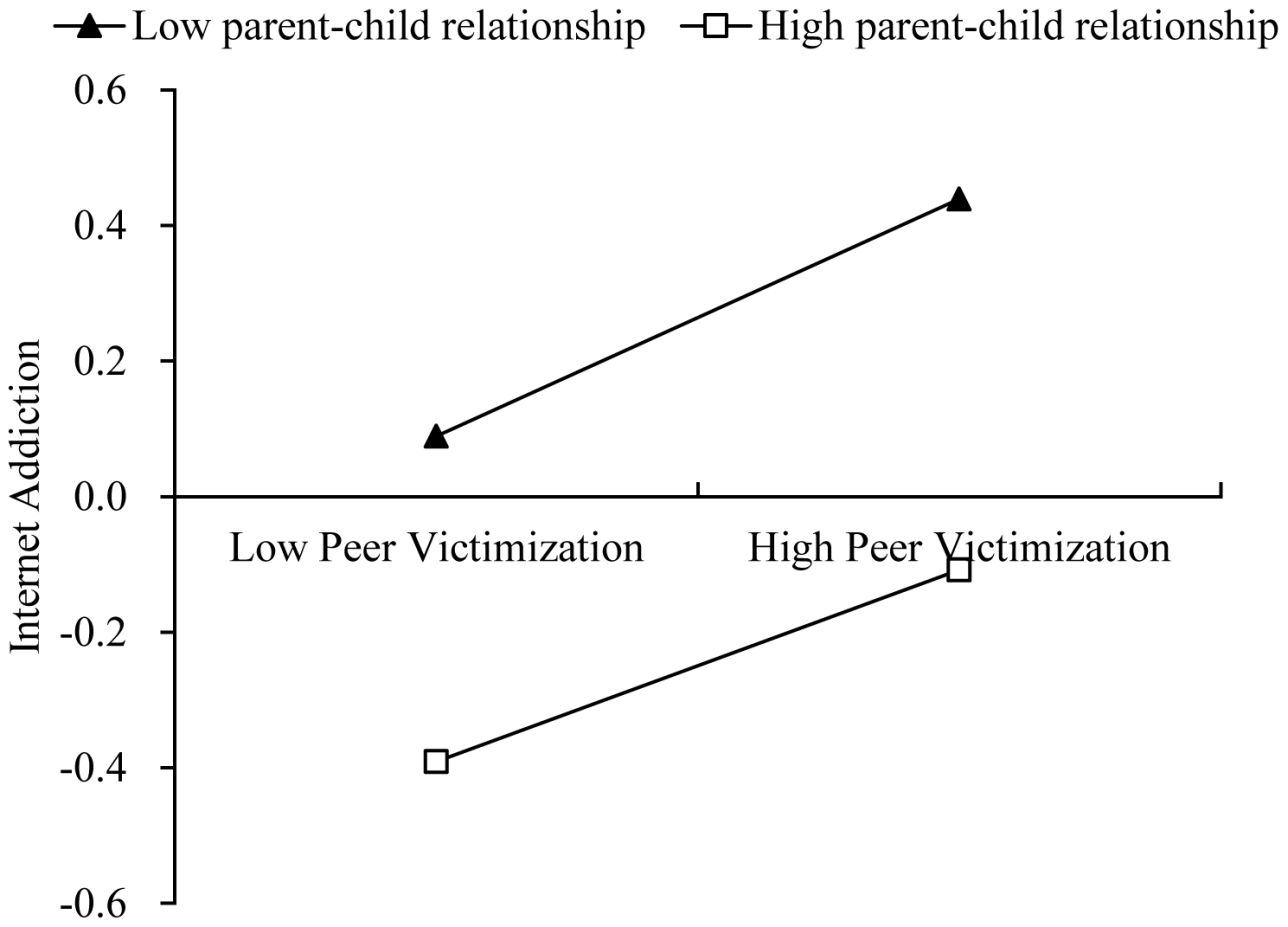
The association between peer victimization and IA conditioned on the levels of PCR. The standardized values of IA were presented near the Y-axis. IA, Internet addiction; PCR, parent–child relationship.

PCR moderated the direct path and the second half of the intermediary process (Table 2). The buffer effect on the first half of the mediating process was not significant (*β* = 0.001, SE = 0.004, 95% CI = [−0.007, 0.009]). However, the interaction between PCR and peer victimization in predicting internet addiction was significant (*β* = −0.017, SE = 0.004, 95% CI = [−0.025, −0.008]). Namely, the direct impact of peer victimization on internet addiction was moderated by PCR. The results of Johnson-Neyman showed that the regression coefficients of peer victimization on internet addiction were significantly greater than zero among PCR ranges of −3.239 ~ 2.469. The regression coefficient decreased with an increase in the PCR quality. In other words, along with the PCR quality increased, the negative effects of peer victimization on Internet addiction showed a downward trend (Figure 2).

**Table 2:** Testing the moderating effect of parent-child relationship on the relationship between peer victimization and internet addiction via subjective well-being.

Variable	Outcome variable: internet addiction			Outcome variable: subjective well-being			Outcome variable: internet addiction		
	*β*	*t*	95% CI	*β*	*t*	95% CI	*β*	*t*	95% CI
Gender	−0.213	−59.084^***^	[−0.412, −0.441]	0.120	1.603	[−0.003, 0.027]	−0.406	−58.458^**^	[−0.392, −0.419]
Peer victimization	0.265	59.130^***^	[0.256, 0.274]	−0.268	−60.226^***^	[−0.277, −0.259]	0.158	34.628^***^	[0.149, 0.167]
Parent-child Relationship							−0.257	−54.788^***^	[−0.266, −0.248]
Parent-child Relationship * Peer victimization							−0.017	−3.793^**^	[−0.025, −0.008]
Subjective Well-being							−0.051	−12.094^***^	[−0.059, −0.043]
Parent-child Relationship * Subjective Well-being							−0.034	−8.319^***^	[−0.042, −0.026]
Socioeconomic Status	−0.102	−27.977^***^	[−0.109, −0.095]	0.161	42.845^***^	[0.154, 0.169]	−0.055	−15.175^***^	[−0.062, −0.048]
*R* ^2^	0.151			0.107			0.213		
*F*	3309.234^***^			2129.193^***^			2274.363^***^		

^**^p<0.01; ^***^p<0.001.

**Figure 3:**
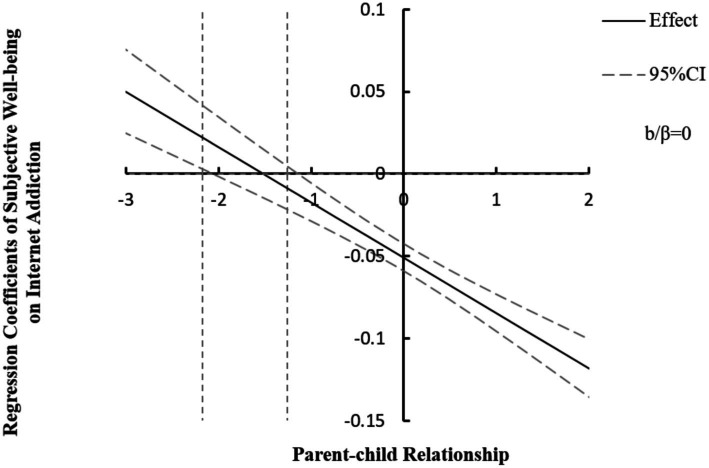
The association between subjective well-being and IA conditioned on the levels of PCR. The standardized values of PCR were showed near the x-axis. The regression coefficients of subjective well-being on IA were presented near the Y-axis. The values of the 95% confidence intervals formed two upper and lower curves. The middle line denotes the values of point estimation. The boundary lines of *p* = 0.05 were represented by two vertical dotted lines. The horizontal dotted line indicated that the regression coefficient was zero, which was overlapped with the horizontal axis. IA, Internet addiction; PCR, parent–child relationship.

In addition, interactions between subjective well-being and PCR was negatively related to Internet addiction (*β* = −0.034, SE = 0.004, 95% CI = [−0.042, −0.026]), suggesting that the impact of subjective well-being on IA was dependent on the levels of PCR. However, the results indicated the presence of a statistically significant inflection point within the observed ranges of PCR with the method of Johnson-Neyman. In PCR ranges of −0.956 to 2.469, based on simple slope tests, the regression coefficients of subjective well-being on Internet addiction were significant and the buffer effect was smaller than zero. However, in PCR ranges of −2.066 to −1.145, the regression coefficients of subjective well-being on Internet addiction were not significant and the 95% confidence intervals contained zero. In PCR ranges of −3.239 to −2.097, the regression coefficients of subjective well-being on Internet addiction attained statistical significance and the buffer effect was bigger than zero. Namely, the negative effect of subjective well-being on Internet addiction was reversed to positive. The results indicated that the buffer effects of subjective well-being on Internet addiction appeared uptrend as the PCR quality increased (Figure 3).

## 4. Discussion and conclusion

We established a moderated mediation model between peer victimization and IA with Chinese children that was mediated by subjective well-being, conditioned on PCR. The results revealed that children being bullied were more susceptible to Internet addiction, which corresponded with the results of previous studies (Hsieh et al., 2019; Zhai et al., 2019). We also found that the direct and indirect links between peer victimization and Internet addiction were moderated by parent–child relationship.

### 4.1. The mediating effect of subjective well-being

Our findings revealed that peer victimization was negatively related to subjective well-being, which in turn predicted internet addiction. It is consistent with the existing literature (Diener and Diener, 1996; Agbaria and Bdier, 2020; Yubero et al., 2021), implying that subjective well-being serves as a “bridge” between peer victimization and compulsive internet use. Peer victimization, closely related to one’s emotional and cognitive evaluation, led to a broad-rang of short-term and long-term behavioral and emotional problems (Yubero et al., 2021), such as low life satisfaction (Stepanikova et al., 2010) and mood disorders (Schoeler et al., 2018; Tripathi, 2018). Children with low levels of subjective well-being exhibit non-adaptive social skills (e.g., aggression) and have less satisfying relationships (Qutaiba and Tamie, 2010). They also respond more slowly to negative events leading to more interpersonal conflicts and negative emotions (Diener and Diener, 1996; Imaginário et al., 2013). In accordance with Psychological Decompensation Hypothesis (Mauss et al., 2007), those with lower subjective well-being in real life may be more probably immersed themselves online to find satisfaction and alleviate negative emotions. Hence, our results reveal that positive subjective well-being is a potential protective factor against compulsive internet use associated with peer victimization.

### 4.2. The moderating effect of parent–child relationship

This study further suggested that PCR moderated the direct and the latter pathway of the mediating process though subjective well-being as a mediator. First, the findings revealed that the impact of peer victimization on IA depended on the PCR quality. Supportive PCR reflects fewer detrimental effects on Internet addiction from peer victimization. The results confirm the theory of the protective model of resilience. The higher the PCR level, the better children’s social competence (Lindsey et al., 2010). With supportive relationships with their parents, children have more opportunities to develop coping mechanisms (under parental guidance) to resolve conflicts, communicate with others, obtain effective sources of support, alleviate symptoms of stress, and build a sense of security outside the home (Bowes et al., 2010; Acar et al., 2019), thereby decreasing the probability and severity of peer victimization (Benetti and Kambouropoulos, 2006). Consequently, although they may be bullied by their peers, children’s relationships could meet their social needs to interact with others (e.g., siblings or friends) without resorting to the cyberspace (Bowes et al., 2010; Kendrick et al., 2012), thus, IA is avoided. The findings demonstrated that PCR could provide adequate protection against Internet addiction following peer victimization. Hence, intervention programs targeting the reduction of conflicts between parents and children, and improvement in the intimacy between them, may be more beneficial for children to improve their peer relationships and to meet their social needs of interacting with others, resulting in a shift away from Internet addiction.

The latter part of the intermediate process was contingent on the PCR quality, whereas the specific pattern of this moderating effect warrants attention. Specifically, the negative impact of subjective well-being on IA only strengthened with a rising PCR when the levels of PCR is large enough (within the range of −0.956 to 2.469). However, when the PCR quality is very small (within the range of −3.239 to −1.145), the negative effects of subjective well-being on Internet addiction is non-significant or turns into positive effects. This may be because children who have suffered from peer victimization have difficulty mitigating the effects of previously established poor subjective well-being (Savahl et al., 2019). These children often make poor emotional and cognitive evaluations (Diener et al., 2010). Because of adopting non-adaptive coping strategies, they have difficulty in processing adverse events and solving problems, which, in turn, lead them to experience more negative emotions and dissatisfaction with their lives. Moreover, to tackle persistent negative emotions and feelings, the likelihood of children resorting to the internet and becoming addicted has greatly increased (Agbaria and Bdier, 2020). Thus, it cannot strengthen the negative effects of low subjective well-being on children’s Internet addiction until PCR levels are large enough. Children who have a high quality of PCR are more likely to regulate poor emotions consciously, use restructuring coping strategies, interact with peers using adaptive social skills, and receive efficacious social support, thereby increasing the impacts of subjective well-being on Internet addiction (Brumariu, 2015).

Out of expectation, the interaction between PCR and peer victimization cannot effectively predict subjective well-being. That is, the first phase of mediation cannot be moderated by PCR. This finding implies that individuals who experience peer victimization are more likely to develop low levels of self-evaluation, irrespective of whether their parent–child relational quality is high or low. That is, the adverse effects of peer victimization on subjective well-being may be fundamental and irreversible. Peer victimization is detrimental to one’s self-concept, leading individuals to have negative evaluations about themselves and their lives, result in concerns related to society, and develop cognitive anxiety (Klomek et al., 2008). Individuals with lower subjective well-being are more likely addicted to the internet (Suresh et al., 2018). Thus, whether children have high or low levels of PCR, the vicious cycle is hard to break.

## 5. Limitations and directions for future research

Although this study provides new insights into protective factors preventing compulsive internet use following peer victimization, we should also consider some limitations. First of all, the sample was limited to elementary school students from various regions in China and the context of all participants was identified as eastern cultures. It is not ascertain whether the findings can be generalized to students from other education levels in China or students from other cultures. In addition, participants with intellectual disability were not taken into consideration yet in this study. These individuals, due to their lower intellectual abilities, are often being bullied at school. The influence mechanism of their bullying experiences on Internet addiction may differ from intellectually normal individuals. Future studies should recruit a larger and more diverse sample for direct comparison across education levels, adopt students from other cultures or even intellectual disability ones. It would be appropriate to replicate the moderated mediation model in other settings to increase the validity of the findings. Second, the data were primarily based on self-reported questionnaires. Reporting bias may thus be more likely to occur in data collection. As such, instead of relying solely on self-report, multiple informants (e.g., teachers, peers, parents) should be considered in future research to replicate the results. Third, participants was a convenience sample in this study, which includes all fourth-grade students in Zhengzhou and all children in grade four from 50 schools in 10 districts of Shijiazhuang. Although the quantity of participants was 65,868, the sample is not designed as a random sampling. Hence, future studies should employ rigorous random sampling design to ensure that the findings can represent the entire situation (e.g., both urban and country students) in China. Fourth, it is difficult to infer causality between the analyzed variables because the current study is cross-sectional in design. Future longitudinal or experimental studies are needed to begin exploring possible causal links between variables. Fifth, in this study, the missing rate for family socioeconomic status is up to 16.4%. That is 10, 848 children. The reason maybe that indicators of family economic status are not well understood by children, such as household possession, parents’ occupation. Family economic status has a significant impact on children’s learning resources, academic adaptation, self-efficacy, and parent–child interaction (Dong et al., 2021). Thus, future studies should design more straightforward questions to reflect indicators of family socioeconomic status and to verify the validity of the model.

In conclusion, our results emphasize the significance of family factors in intervention programs to alleviate IA for children who have experienced peer victimization. For bullied children, supportive relationships at home buffer against the risk of emergent and persistent of Internet addiction. Hence, parents and teachers should try their best to enhance their relationships with children, as well as to help children improve their emotional regulation skills, and develop coping mechanisms to deal with conflicts and upsetting emotions. This will enable them to manage various stressful situations and adverse emotions when they experience peer victimization. We also found that subjective well-being is a protective factor to intervene Internet addiction related to peer victimization, demonstrating the mechanism of how IA is influenced by victimization. As such, supportive PCR and positive self-evaluations should be reinforced among children to reduce the risk of compulsive internet use stemming from victimization experiences.

Further, children with internet addiction may have unsatisfied needs in certain aspects of their life (Suler, 1999; Zhou et al., 2017). For example, the sense of belonging and security for children who have addicted to the internet related to victimization (Suler, 1999; Liu et al., 2016). Parents should communicate with their children frequently and pay more attention to psychological matters such as whether they have conflicts with friends, experience strong negative emotions or have academic pressure. It is important to provide psychological guidance to children. Spending time with children engaging in outdoor activities such as running, playing sports, and playing chess can help them develop more interests and enrich their inner world, leading them to disengage from the internet.

## Data availability statement

The raw data supporting the conclusions of this article will be made available by the authors, without undue reservation.

## Ethics statement

The studies involving humans were approved by the Institutional Review Board of Beijing Normal University. The studies were conducted in accordance with the local legislation and institutional requirements. Written informed consent for participation in this study was provided by the participants’ legal guardians/next of kin.

## Author contributions

PZ, JL, and WH designed the study, analyzed data, and worked on manuscript preparation. JL was the grant applicant and project leader, overall management, data analysis and interpretation. JPC, JXC, FC, and CZ assisted with data analysis and manuscript preparation. ZW and JXC done the methodology and data analysis. All authors contributed to the article and approved the submitted version.
